# Tendon and Cytokine Marker Expression by Human Bone Marrow Mesenchymal Stem Cells in a Hyaluronate/Poly-Lactic-Co-Glycolic Acid (PLGA)/Fibrin Three-Dimensional (3D) Scaffold

**DOI:** 10.3390/cells9051268

**Published:** 2020-05-20

**Authors:** Maria C. Ciardulli, Luigi Marino, Joseph Lovecchio, Emanuele Giordano, Nicholas R. Forsyth, Carmine Selleri, Nicola Maffulli, Giovanna Della Porta

**Affiliations:** 1 Department of Medicine, Surgery and Dentistry, University of Salerno, Via S. Allende, 84081 Baronissi (SA), Italy; mciardulli@unisa.it (M.C.C.); lmarino@unisa.it (L.M.); cselleri@unisa.it (C.S.); n.maffulli@qmul.ac.uk (N.M.); 2 Department of Electrical, Electronic and Information Engineering “Guglielmo Marconi” (DEI), University of Bologna, Via dell’Università 50, 47522 Cesena (FC), Italy; joseph.lovecchio@unibo.it (J.L.); emanuele.giordano@unibo.it (E.G.); 3 Guy Hilton Research Centre, School of Pharmacy and Bioengineering, Keele University, Stoke-on-Trent, Staffordshire ST4 7QB, UK; n.r.forsyth@keele.ac.uk; 4 Centre for Sport and Exercise Medicine, Queen Mary University of London, Barts and The London School of Medicine, London E1 4NL, UK; 5 Department of Industrial Engineering, University of Salerno, Via Giovanni Paolo II, 84084 Fisciano (SA), Italy

**Keywords:** hBM-MSCs, cytokines, tenogenic markers, cyclic strain, 3D microenvironment, PLGA carriers, bioreactor

## Abstract

We developed a (three-dimensional) 3D scaffold, we named HY-FIB, incorporating a force-transmission band of braided hyaluronate embedded in a cell localizing fibrin hydrogel and poly-lactic-co-glycolic acid (PLGA) nanocarriers as transient components for growth factor controlled delivery. The tenogenic supporting capacity of HY-FIB on human-Bone Marrow Mesenchymal Stem Cells (hBM-MSCs) was explored under static conditions and under bioreactor-induced cyclic strain conditions. HY-FIB elasticity enabled to deliver a mean shear stress of 0.09 Pa for 4 h/day. Tendon and cytokine marker expression by hBM-MSCs were studied. Results: hBM-MSCs embedded in HY-FIB and subjected to mechanical stimulation, resulted in a typical tenogenic phenotype, as indicated by type 1 Collagen fiber immunofluorescence. RT-qPCR showed an increase of type 1 Collagen, scleraxis, and decorin gene expression (3-fold, 1600-fold, and 3-fold, respectively, at day 11) in dynamic conditions. Cells also showed pro-inflammatory (IL-6, TNF, IL-12A, IL-1β) and anti-inflammatory (IL-10, TGF-β1) cytokine gene expressions, with a significant increase of anti-inflammatory cytokines in dynamic conditions (IL-10 and TGF-β1 300-fold and 4-fold, respectively, at day 11). Mechanical signaling, conveyed by HY-FIB to hBM-MSCs, promoted tenogenic gene markers expression and a pro-repair cytokine balance. The results provide strong evidence in support of the HY-FIB system and its interaction with cells and its potential for use as a predictive in vitro model.

## 1. Introduction

Tissue engineering strategies for tendon healing and regeneration are designed to improve existing therapies or provide new treatment possibilities. Three-dimensional (3D) bioengineered systems have the potential to promote our understanding of the physiopathology of tendinopathy and the role of stem cells in tendon regeneration. In this sense, 3D scaffold design and fabrication coupled to specific bioreactor arrangements could develop highly predictive 3D in vitro culture and differentiation systems to explore cell behaviors in response to defined external biochemical and mechanical stimuli [1]. The 3D scaffold provides a model of fidelity via its provision of a microenvironment with defined stiffness and elastic modulus as well as the necessary surfaces for cell attachment [2,3,4]. 

Detailed understanding of cell behavior when incorporated into specific biomaterials allows to develop designs with specific functionalization. These functionalization may, for instance, stimulate local stem cells, attract specific circulating nucleated blood cells, such as macrophages, and induce their polarization into M2 phenotype to accelerate tissue regeneration and healing following the biomaterials in vivo implantation [5]. For example, human Mesenchymal Stem Cells (hMSCs) are largely used in tissue engineering strategies, and their immune-modulatory activity in the development of tendon pathologies have been explored, but the precise mechanisms involved remain undetermined [6,7,8,9,10]. Neutrophils and macrophages infiltrate injured tendons, potentially interacting with MSCs and stimulating cytokine release at the site of repair and promoting degradation of the extracellular matrix (ECM), inflammation, apoptosis, and, in the later stages of acute tendon healing, they release anti-inflammatory cytokines to alleviate inflammation and promote tendon remodeling [11,12,13].

Among the biomaterials described for tendon tissue engineering [14], a promising emerging strategy is the use of a complex biomimetic matrix with a hydrogel component and extracellular matrix mimicking properties [15,16]. Tenocyte precursors can be harvested from different sources, including periosteum [17,18], bone marrow [19,20,21], tendon [21,22], and adipose tissue [21,23]. To overcome the intrinsic poor mechanical properties of the hydrogel, they can be merged with more force resistant biopolymers. Cells and biomaterials alone are not sufficient to achieve optimal levels of differentiation and matrix organization. Mechanical stimulation has a key role in tenogenic differentiation induction [19]. Scaffolds are therefore required to display an appropriate elastic behavior to deliver strain [24] or compression [25] inputs. Strain is a tenogenic differentiation signal [26,27,28,29], and several bioreactors have been used to impart tenogenic mechanical stimuli to cells in culture [19,30,31,32,33,34,35]. For example, Rinoldi et al. designed and fabricated 3D multilayered composite scaffolds, where an electrospun nanofibrous substrate was coated with a thin layer of GelMA-alginate composite hydrogel carrying MSCs. MSCs were subsequently differentiated by the addition of bone morphogenetic protein 12 (BMP-12) and, to mimic the natural function of tendons, the scaffolds were mechanically stimulated using a custom-built bioreactor [34]. Grier et al. described an aligned collagen-glycosaminoglycan scaffold able to enhance tenogenic differentiation of MSCs via cyclic tensile strain within a bioreactor, in the absence of growth factor supplementation [36]. Another protocol, proposed by Youngstrom et al., promoted tenogenic differentiation of MSCs cultivated on decellularized tendon scaffolds with the application of 3% cyclic strain for one hour per day for 11 days [31]. Additionally, several growth factors and other small molecules can stimulate transcriptional activation of genes involved in tenogenic differentiation [15,37,38]. Growth Differentiation Factor 5 (GDF-5), for instance, induces the expression of genes linked to the neo-tendon phenotype [39,40,41].

Tendinopathies associated with physical activity and age-related degeneration are a major medical issue [23], and recent healing and regeneration studies include the use of human Bone Marrow Mesenchymal Stem Cells (hBM-MSCs) [42,43,44]. hBM-MSCs are a multipotent population present in bone marrow that can be readily differentiated in vitro [45,46] into cells of three mesodermal lineages, namely adipocytes, chondrocytes and osteoblasts under appropriate conditions [47,48,49,50]. MSCs-based therapies include direct transplantation of MSCs populations, growth factor-loaded scaffolds for local MSCs recruitment or implantation of scaffolds containing in vitro culture-expanded MSCs populations [51,52].

We previously described an engineered multiphase three-dimensional (3D) scaffold as an in vitro model for tendon regeneration studies. The multiphase 3D construct was totally absorbable and consisted of a braided hyaluronate elastic band merged with a fibrin hydrogel containing hBM-MSCs and poly-lactic-co-glycolic acid nano-carriers (PLGA-NCs) themselves loaded with human Growth Differentiation Factor 5 (hGDF-5) [53]. In that work, the PLGA nano-carriers were transient scaffold components to ensure sustained and controlled delivery of hGDF-5 with benefits beyond those associated with standard culture medium supplementation [53,54]. The study reported an early tenogenic commitment of hBM-MSCs after three days of cultivation under dynamic conditions. 

In the present study, we describe the use of the same scaffold (named HY-FIB here) to investigate the effect of the 3D environment on hBM-MSCs for 11 days with or without mechanical stimulation and in the absence of any specific biochemical differentiation signal. HY-FIB was assembled with hBM-MSCs as previously described [53] including PLGA-NCs stratified within the 3D fibrin structure. Importantly, the PLGA-NCs carried an inactive form of human Growth Differentiation Factor 5 (ihGDF-5) enabling the overall 3D scaffold structure to be safely evaluated with or without mechanical input. Gene expression of type 1 Collagen, decorin, scleraxis, and tenomodulin were considered; type 3 collagen was also monitored, as negative control. Histology and quantitative immunofluorescence were used to monitor cell behavior and their interaction with the synthetic extracellular matrix. Moreover, to understand whether HY-FIB microenvironment configuration would stimulate any cell inflammation responses, the cells expression of cytokine markers was also monitored, including pro-inflammatory cytokines and anti-inflammatory ones. The results provide strong evidence that HY-FIB environment plus mechanical signaling, promoted tenogenic markers expression, collagen production and better pro-repair cytokine balance by hBM-MSCs. 

## 2. Materials and Methods

### 2.1. hBM-MSCs Isolation and Harvesting

Human bone marrow mesenchymal stem cells (hBM-MSCs) were obtained from the bone marrow of three independent healthy donors (age 36, 38, 44 years). The donors gave written informed consent in accordance with the Declaration of Helsinki to the use of their filter residual bone marrow aspirate for research purposes, with approval from the University Hospital of San Giovanni di Dio e Ruggi d’Aragona (Salerno, IT). Review Board authorization number: (24988 achieved on April 9, 2015). 

Briefly, total bone marrow aspirate was directly seeded at a concentration of 50,000 total nucleated cells/cm^2^ in T75 plastic flask in Minimum Essential Medium Alpha (α-MEM) supplemented with 1% Glutagro^TM^, 10% Fetal Bovine Serum (FBS), and 1% Pen/Strep and incubated at 37 °C in 5% CO_2_ atmosphere and 95% relative humidity [55]. After 72h, non-adherent cells were removed by medium change, and the adherent cells were further fed twice a week with new medium. On day 14, colonies of adherent hBM-MSCs were detached and re-seeded at 4000 cells/cm^2^ in the same culture conditions. Once the cell cultures reached 70–80% confluence, cells were detached using 0.05% trypsin-0.53mM EDTA and washed with PBS 1× (Corning Cellgro, Manassas, VA, USA), counted using Trypan Blue (Sigma-Aldrich, Milan, IT) and subcultured at a concentration of 4 × 10^3^ cells/cm^2^. Flow Cytometry analysis was performed on hBM-MSCs obtained at Passage 1 examining levels of CD90, CD105, CD73 CD14, CD34, CD45, and HLA-DR expression (Miltenyi Biotec, DE). 

### 2.2. ihGDF-5 Effect on hBM-MSCs

These sets of experiments were performed to assure the absence of any effect of inactivated human GDF-5 (ihGDF-5, Cloud-Clone Corp., USA) on both tenogenic markers stimulation and cytokines expression by hBM-MSCs. Cells were seeded on coverslips in 12 well plates at a concentration of 4 × 10^3^ cells/cm^2^. Once the cultures reached 60% confluence, cells were treated with either 1.6 ng/mL or 100 ng/mL of ihGDF-5. Cells were fed twice a week with new medium and fresh ihGDF-5 supplementation for up to 16 days. Untreated cells for matched time-points studied were used for control purposes. Passage 3 cells were seeded in the 3D environment (~8 × 10^5^ cells/mL) and were fed twice a week with new medium, without any growth factor added. 

PLGA carriers were not tested because they cannot be supplemented to the cells planar monolayer culture, indeed, in static conditions their sedimentation on cells reduced oxygen exchange and prevented cells survival (data not shown).

### 2.3. Immunofluorescence and Immunohistochemical Assays

Cells were fixed with 3.7% formaldehyde for 30 min at room temperature (RT) followed by permeabilization with 0.1% Triton X-100 for 5 min and blocking with 1% Bovine Serum Albumine (BSA) for 1h. For type 1 and type 3 Collagen staining, cells were incubated overnight at 4 °C with a mouse monoclonal anti-type 1 Collagen antibody (1:100, Sigma-Aldrich) and a rabbit polyclonal anti-type 3 Collagen antibody (1:100, Santa Cruz Biotechnology). Following incubation with the primary antibody, cells were incubated for 1h at RT with the DyLight 649 anti-mouse IgG (1:500, BioLegend, CA) and the Alexa FluorTM 488 goat anti-rabbit IgG (1:500; Thermo Fisher Scientific, USA). Cell nuclei were stained with DAPI solution (1:1000) for 5 min. Images acquisition was at 20× magnification on a fluorescent microscope (Eclipse Ti-E Inverted Microscope; NIKON Instruments Inc., USA). 

For 3D scaffold immunohistochemical analysis, slices were permeabilized with 0.1% Triton X-100 for 5 min, and non-specific staining blocked with 1% BSA for 1h at RT. For type 1 Collagen staining, slices were incubated overnight at 4 °C with a rabbit polyclonal anti-type 1 Collagen antibody (1:200, AbCam). Following incubation with the primary antibody, slices were incubated for 1h at RT with Alexa Fluor^TM^ 488 goat anti-rabbit IgG (1:400, Thermo Fisher Scientific, USA) antibody. Subsequently, cell nuclei were stained with DAPI solution (1:1000) and incubated for 5 min. Images were acquired as described above. Image quantification was performed using image analysis software (ImageJ, National Institutes of Health, USA) [56,57] by measuring the red and green areas where type 1 and type 3 collagen, respectively, are expressed. A minimum of 10 image fields was used for the image analysis at each time point. Signal intensity at each time point was normalized by the cell number (e.g., by amount of cell nuclei revealed by DAPI staining).

Sirius red staining was performed using the Picrosirius Red Stain Kit (Polysciences, Inc., USA). Sections of 15 μm of thickness were stained in hematoxylin for 8 min, then washed in water for 2 min. The sections were dipped into phosphomolybdic acid for 2 min, then washed in water for 2 m. Then they were dipped into Picrosirius Red F3BA Stain for 60 min and dipped into HCl 0.1M solution for 2 min. The sections were dehydrated in increasing ethanol gradient solutions (70–75–95–100%) and finally dipped into xylene for 5 min. Eukitt medium was used to mount the samples.

### 2.4. RNA Isolation and Gene Expression Profile by Quantitative Reverse Transcription PCR (RT-qPCR)

Total RNA was extracted from hBM-MSCs seeded into the 3D construct of each experimental group using QIAzol^®^ Lysis Reagent (Qiagen, DE), chloroform (Sigma-Aldrich, Milan, IT) and the RNeasy Mini Kit (Qiagen, DE). For each sample, 300 ng of total RNA was reverse-transcribed using the iScriptTM cDNA synthesis kit (Bio-Rad, Milan, IT). Relative gene expression analysis was performed in a LightCycler^®^ 480 Instrument (Roche, IT), using the SsoAdvancedTM Universal SYBR^®^ Green Supermix (Bio-Rad) with the validated primers for COL1A1, COL3A1, DCN, IL-1β, IL-6, IL-10, IL-12A, SCX-A, TGF-β1, TNF, and TNMD (Bio-Rad), and following MIQE guidelines [58]. Amplification was performed in a 10 μL final volume, including 2 ng of complementary DNA (cDNA) as template. Specificity of the formed products was addressed via melting curve analysis. Triplicate experiments were performed for each condition explored, and data were normalized to glyceraldehyde-3-phosphate dehydrogenase (GAPDH) expression (reference gene), applying the geNorm method [59] to calculate reference gene stability between the different conditions (calculated with CFX Manager software; M <0.5). Fold changes in gene expression were determined by the 2^−ΔΔCp^ method, and are presented as relative levels versus hBM-MSCs just loaded within the HY-FIB system. 

### 2.5. PLGA-NCs Fabrication, Size, Morphology, and ihGDF-5 Release Profile

PLGA nano-carriers (PLGA-NCs) were obtained using Supercritical Emulsion Extraction (SEE) technology enabling rapid polymer NCs production from multiple emulsions via dense gas extraction of the oily phase organic solvent utilizing a countercurrent packed tower operating in continuous mode [60]. In detail, ihGDF-5 (Cloud-Clone Corp., USA) was dissolved into 1% (*w*/*v*) human serum albumin (hSA; Sigma-Aldrich, Milan, IT) containing 0.06% polyvinyl alcohol (PVA). Human serum albumin (hSA) was included as an ihGDF-5 stabilizer. This solution was added to the oily phase formed in an Ethyl Acetate (EA, purity 99.9%) and PLGA (RG 504H, 38,000–54,000 kDa, Evonik, DE) at 5% (*w*/*w*) solution. All emulsions were processed immediately after their preparation. 

SEE technology operative pressure and temperature conditions in the high-pressure column were set at 8 MPa and 38 °C, respectively, with a dense gas flow of Carbon Dioxide (CO_2_) set at 1.4 kg/h with Liquid/Gas ratio of 0.1 (*w*/*w*) [61]. Carrier suspensions were collected at the bottom of the extraction column, washed, and lyophilized. Each run allowed the recovery of 98% of the loaded biopolymer. Empty and loaded NCs were produced using the same process conditions.

Carrier particle size distributions (PSDs) were measured using a laser granulometer (mod. Mastersizer S; Malvern Instruments Ltd., Worcestershire, UK), based on dynamic light scattering (DLS). Sizes are expressed as volume mean size (MS) with standard deviation (SD) in nanometers (nm). The shape and morphology of the PLGA-NCs were investigated by field emission-scanning electron microscopy (FE-SEM; mod. LEO 1525; Carl Zeiss, Oberkochen, D). Samples were placed on a double-sided adhesive carbon tape previously glued to an aluminum stub and coated with a gold film (250 A thickness) using a sputter coater (mod.108 A; Agar Scientific, Stansted, UK). 

ihGDF-5 release profile was monitored in vitro from 20(±0.3) mg of carriers suspended in 1mL of α-MEM, placed in an incubator at 37 °C, and stirred continuously at 1× *g*. Every 24 h, samples were centrifuged at 160× *g* for 10 min and the supernatant completely removed and replaced with fresh media to maintain sink conditions. Released ihGDF-5 concentrations from collected samples were then measured with an Enzyme Linked Immunosorbent Assay (ELISA, Cloud-Clone Corp., USA). Release experiments were performed in triplicate (n = 3), and the curve describing the mean profile calculated as ng/g (protein released/PLGA-NCs) versus time.

### 2.6. HY-FIB Preparation and Characterization

For each sample, a mixture of 50 mg/mL fibrinogen from human plasma (Sigma-Aldrich, Milan, IT), 15,600 U/mL aprotinin (Sigma-Aldrich, Milan, IT), and α-MEM (Corning, NY, USA) supplemented with 10% FBS (referred to as growing media, GM) was added at a 1:1:1 ratio to 100 mg of PLGA-NCs (ihGDF-5 loading: 350 ng/g) and, then, to an average of 8 × 10^5^ cells. A homogeneous cells/PLGA-NCs/fibrinogen suspension was then embedded into a mold (30 × 20 × 4.5 mm) where the braided band had been previously positioned. Free ends were left to enable HY-FIB fixing into the bioreactor. Upon addition of 100 U/mL thrombin (Sigma-Aldrich, Milan, IT), the mold was placed in a 37 °C humidified incubator for 30 min to allow fibrin polymerization. When the hydrogel was formed, the band was entrapped inside a uniformly distributed hydrogel. The construct was then transferred from the mold to either a standard polystyrene culture plate or to the bioreactor culture chamber, each containing 30 mL of the culture media, and placed in an incubator at 37 °C in a 5% CO_2_ atmosphere and 95% relative humidity.

HY-FIB morphology was observed by field emission-scanning electron microscopy (FE-SEM; mod. LEO 1525; Carl Zeiss, Oberkochen, Germany). Samples were fixed in 4% PFA (4 °C, overnight) and then dehydrated by multiple passages across ethanol:water solutions (10 min each) with increasing concentrations of ethanol (10%, 20%, 30%, 50%, 70%, 90%), ending in a 100% dehydrating liquid (3 changes, 10 min each). 

Samples were then lyophilized in a Critical Point Dryer (mod. K850 Emitech, Assing, Rome IT), placed on a double-sided adhesive carbon tape previously glued to an aluminum stub and coated with a gold film (250 A thickness) using a sputter coater (mod.108 A; Agar Scientific, Stansted, United Kingdom) before observation. 

HY-FIB mechanical characterization was performed according to the ASTM 1708 by a dynamometer (CMT 6000 SANS, Shenzen, China) equipped with a 1 kN load cell. The sample was conditioned in Dulbecco’s Modified Essential Medium (DMEM) for 1 h, and then shaped to obtain a specimen with gauge length (Lo) of 22 mm and width (W) of 5 mm. Sample thickness (S) was measured with a thickness gauge brand at three different averaged points. Monoaxial deformation was applied to the sample at a speed of 10 mm/min, and force (F) and elongation (L) during traction were recorded. The elastic modulus and ultimate tensile strength (both expressed in MPa) were calculated from the stress/strain plot. For the immuno-histochemical analysis, at different time points, a portion of HY-FIB was fixed in 4% PFA (4 °C, overnight), cryo-protected in 30% sucrose overnight, mounted in OCT embedding compound, frozen at −20°C and then cut in slices of 10 μm of thickness using a cryostat. The remaining portion of HY-FIB was placed in QIAzol^®^ Lysis Reagent for total RNA extraction.

### 2.7. Dynamic Culture

HY-FIB was clamped at both free ends, one motionless and one sliding (operated by a linear motor actuator) arm, into the bioreactor system culture chamber, described in detail elsewhere [23]. A maximal load, set by pre-tensioning, was relaxed to a minimum value cycling at a pre-determined frequency. In addition, continuous feedback signals provided by strain gauges located onto the fixed arm, allowed the maintenance of a defined load on the scaffold in response to physical system modifications, by automatic adjustment of the pre-tensioning position. 

### 2.8. Finite Element Modeling

Finite Element Modeling (FEM) was implemented by using COMSOL Multiphysics Software (rel. 5.3a^®^) to assess the stress distribution into 3D constructs when a 10% mechanical cyclic strain stimulus is applied. All components were obtained using primitive geometries and Boolean operations. Linear elasticity equations were set as boundary conditions for the scaffold. A sensitivity study of the mesh obtained the most computationally efficient solution. The specific parameters used in the model are listed in Table 1. The simulation considered only the stress within the fibrin 3D environment neglecting any further contribution of the band, cells and PLGA-NCs.

### 2.9. Statistical Analysis

Statistical analysis was performed using GraphPad Prism software (6.0 for Windows). Data obtained from multiple experiments are expressed as mean+/−SD and analyzed for statistical significance using ANOVA test, for independent groups. Differences were considered statistically significant when *p* ≤ 0.05 [63].

## 3. Results

### 3.1. hBM-MSCs Cultivation in 2D Environment

**Figure 1:**
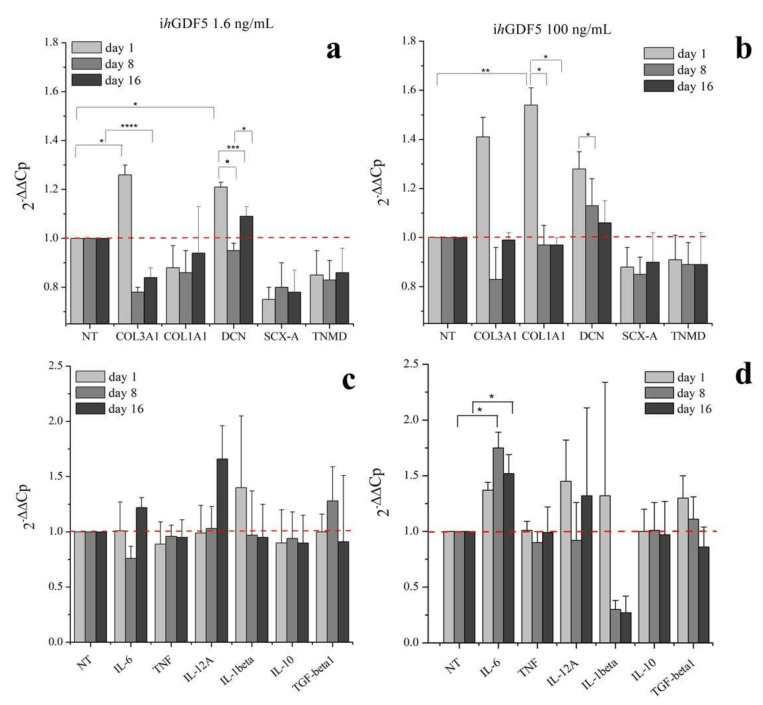
Gene expression profiles for tenogenic markers and pro-inflammatory and anti-inflammatory cytokines by hBM-MSCs treated with 1.6 ng/mL (**a**,**c**) and 100 ng/mL (**b**,**d**) of ihGDF-5 in monolayer 2D culture up to 16 days. mRNA levels of COL1A1, DCN, SCX-A, and TNMD were considered as tenogenic markers and COL3A1 selected as negative ones; pro-inflammatory (IL-6, TNF, IL-12A and IL-1β) and anti-inflammatory (IL-10 and TGF-β1) cytokines were monitored. ihGDF-5 not had significant impact on genes expression, especially at the lower concentration tested. Untreated cells for matched time-points were used as control. * ≤ 0.05; ** <0.01; *** <0.005; **** <0.001 N = 3 (biological replicates); n = 3 (technical replicates).

hBM-MSCs were cultivated in a two-dimensional (2D) monolayer environment with medium supplemented with either 1.6 ng/mL or 100 ng/mL of inactive human Growth Differentiation Factor 5 (ihGDF-5) for up to 16 days. These two concentration conditions (two order of magnitude of difference) were chosen to ascertain absence of any effect of the inactive hGDF-5 form on cells expression of tenogenic markers (COL3A1, COL1A1, DCN, SCX-A and TNMD) and of cytokines (pro-inflammatory: IL-6, TNF, IL-12A, IL-1β; anti-inflammatory: IL-10, TGF-β1) by RT-qPCR (see Figure 1a–d). 

**Figure 2:**
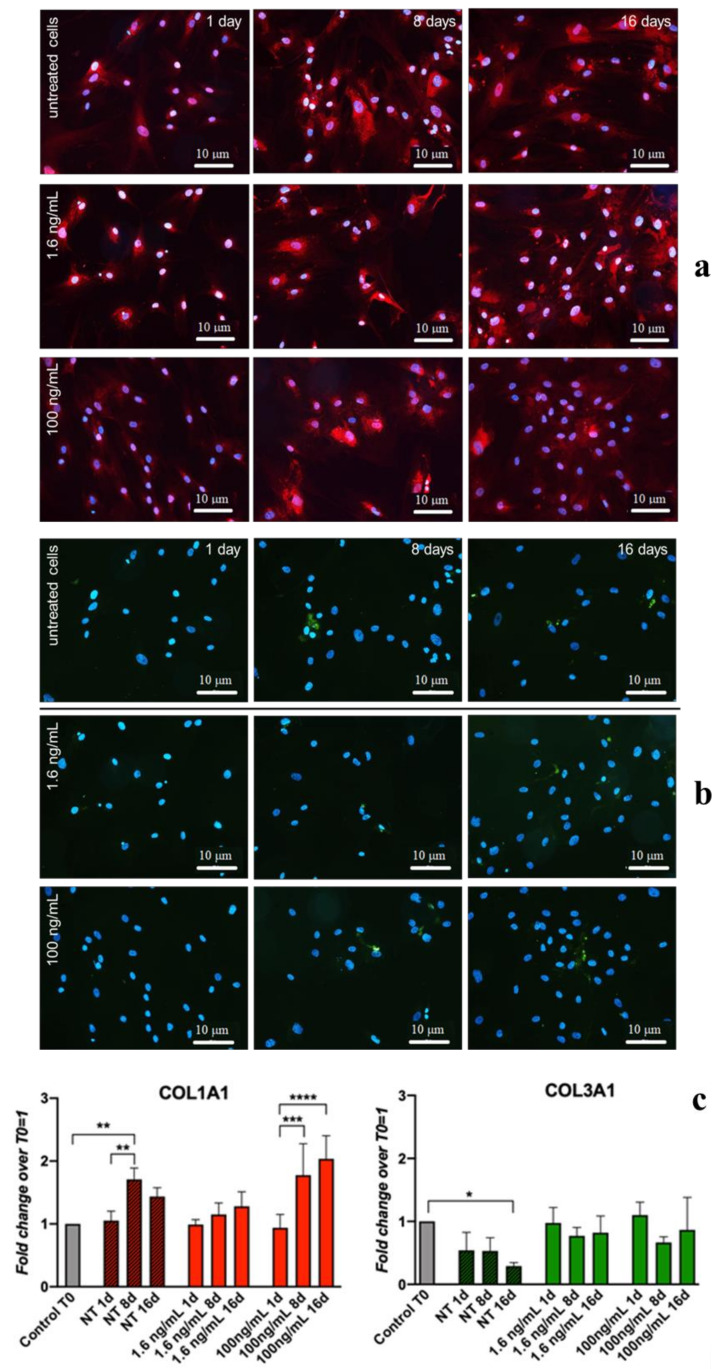
IF and quantitative-IF assays of type 1 Collagen (COL1A1) and type 3 Collagen (COL3A1) monitored along hBM-MSCs treatment with 1.6 ng/ and 100 ng/mL of ihGDF-5 for 16 days. Type 1 collagen was stained in red; type 3 collagen was stained in green; cell nuclei highlighted with DAPI in blue (**a**,**b**). Quantitative signal detection was performed via ImageJ software (**c**). A slight up-regulation of COL1A1 was observed when hBM-MSC were in routine culture for 16 days. Color intensity in each time point was normalized by the cell number. * ≤0.05; * *<0.01; *** <0.005; **** <0.001. n = 10 (image fields for each time point).

Transient and slight, though significant, upregulation of COL3A1 (0.4-fold), DCN (0.2-fold), and COL1A1 (0.5-fold) was observed at Day 1 in cultures supplemented with 100 ng/mL ihGDF-5 (Figure 1b). The reduced dose of 1.6 ng/mL induced low-level transient expression at Day 1 for only COL3A1 (0.3-fold) and DCN (0.2-fold) only (Figure 1a). No significant upregulation was noted for TNF, IL-12A, IL-1β, IL-10, or TGFβ at any time point or ihGDF-5 concentration tested (Figure 1c,d). Compared to controls, IL-6 displayed significant levels of elevation at Days 8 (0.8-fold) and 16 (0.5-fold) with 100 ng/mL ihGDF-5 (Figure 1d). Types 1 and 3 Collagen expression levels were monitored by immunofluorescence during the 16 day culture period, as illustrated in Figure 2a,b. Immunofluorescence quantitative data by image analysis were congruent with RT-qPCR outputs when 1.6 ng/mL ihGDF-5 was supplemented, in this case, both proteins signals were not significantly elevated, compared to untreated cells (see Figure 2c). 

Quantitative image analysis displayed COL1A1 signal increase (1 fold) at Day 16 only with 100 ng/mL ihGDF-5 supplementation. This last data is in contrast with gene expression ones. COL1A1 and COL3A1 are the major components of the extracellular matrix in connective tissues, and their slight up-regulations was reported when hBM-MSC were in routine culture for 16 days [64]. However, in our case, ihGDF-5 seemed not to impact on their production, especially at the lower concentration tested. This preliminary information is important to confirm the inactivity of the biochemical input in regards to the gene expression and proteins that will be monitored in the 3D experiments.

### 3.2. PLGA-NCs Fabrication and ihGDF-5 Controlled Deliver

**Figure 3:**
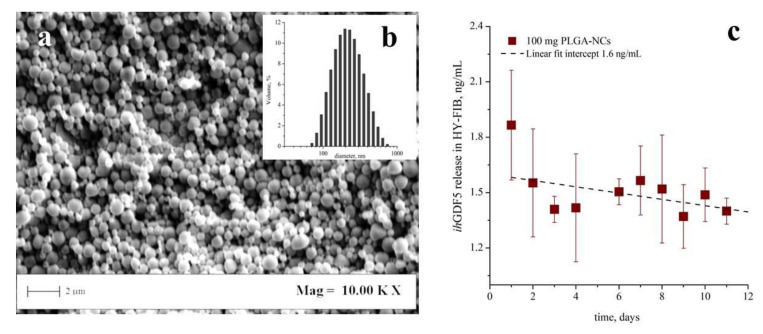
Poly-lactic-co-glycolic-acid (PLGA) transient carriers field emission-scanning electron microscopy (FE-SEM) image, particle size distribution, and ihGDF-5 release profile. FE-SEM images indicated spherical morphology of carriers (**a**); the size distribution set at 230 ± 80 nm their mean size (**b**). Release profiles performed in vitro at 37 °C indicated a ihGDF-5 mean concentration of 1.6 ng/mL/day released from the 100 mg of PLGA-NCs loaded within HY-FIB over 11 days of culture (**c**).

Poly-lactic-co-glycolic-acid nano-carriers (PLGA-NCs) displayed a spherical morphology with a mean size of 230 ± 80 nm (Figure 3a). PLGA-NCs had an ihGDF-5 loading of 350 ng/g and provided a daily released peptide mean concentration of 1.6 ng/mL/day (Figure 3b), when an amount of 100 mg were inserted within HY-FIB over 11 days of culture. As highlighted above, these ihGDF-5 concentration levels did not stimulate sustained impacts on hBM-MSCs gene expression in 2D monolayer culture (see Figure 1a,c). 

Therefore, by excluding any non-specific ihGDF-5 induction (released within the 3D scaffold by the NCs), we could now observe cell behaviors arising from the HY-FIB microenvironment in both static and dynamic conditions.

### 3.3. hBM-MSCs Cultivation in HY-FIB 3D Microenvironment

**Figure 4:**
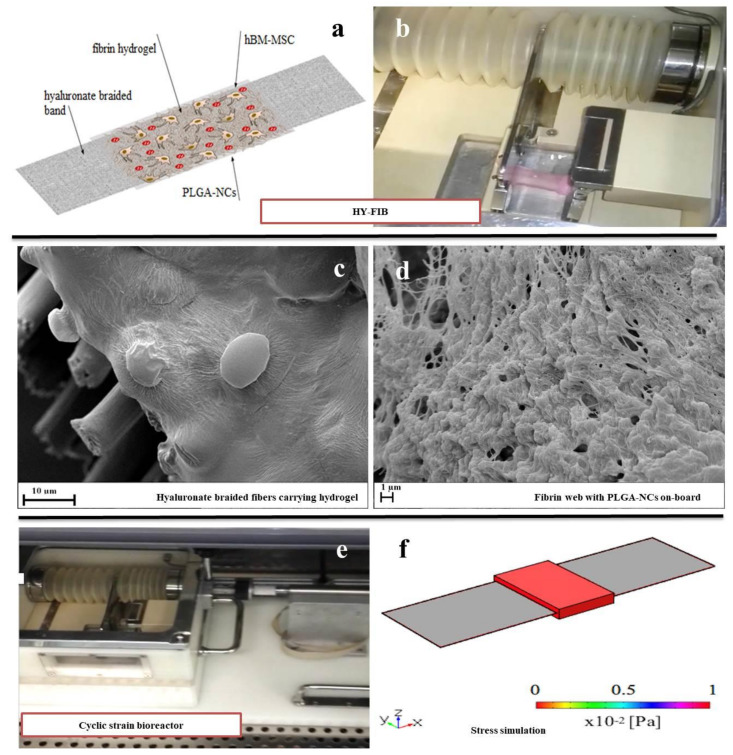
HY-FIB three-dimensional (3D) scaffold features and cyclic strain bioreactor. Schematic HY-FIB representation (**a**) and image of 3D scaffold (**b**)**.** SEM images of hyaluronate braided fibers (10 μm mean diameter size) (**c**) joined to a fibrin web which entrapped both NCs and hBM-MSCs (**d**). Cyclic strain bioreactor (**e**) and in-silico study of stress distribution over HY-FIB upon mechanical strain of 10% (**f**). The simulation involved only the stress of the fibrin 3D environment, neglecting any further contribution.

The HY-FIB assembly featured a braided band (3 × 10cm) joined to a fibrin hydrogel (on a band surface of 6 cm^2^) containing 8 × 10^5^ hBM-MSCs and 100 mg of ihGDF-5/PLGA-NCs. The picture and schematic representation of the 3D system is shown (Figure 4a,b). Field Emission Scanning Electron Microscopy (FE-SEM) images of the scaffold illustrate hyaluronate fibers, embedded within a fibrin hydrogel (Figure 4c), which provided an entrapment surface for both NCs and hBM-MSCs (Figure 4d). 

HY-FIB was exposed to 10% deformation over a 1 Hz frequency for 4 h a day during the dynamic culture experiments via a cyclic strain bioreactor, illustrated in Figure 4e [24]. In greater detail, a HY-FIB braided band was held at one end by a motionless arm and at the other end by a sliding one. Motion was driven by a linear motor and transmitted through the braided band to cells embedded within the fibrin hydrogel. The motionless arm comprises a base, attached to the side wall of the culture chamber, housing the electronic components for load monitoring, and from which extended a cantilevered shelf whose deformation is measured by four strain gauges. The whole system was housed within an incubator to ensure the appropriate CO_2_ gaseous environment to control the pH of the cell culture media and 37 °C operational temperature. 

The stress delivered to the cells immobilized within the system was explored via computational analysis that estimated a mean shear stress value estimated at 9 × 10^−2^ Pa within the fibrin 3D environment (Figure 4f). This order of magnitude of stress value was reported for tenogenic induction [30]; larger deformation for longer times were excluded to focus the study on 3D environment assembled. 

**Figure 5:**
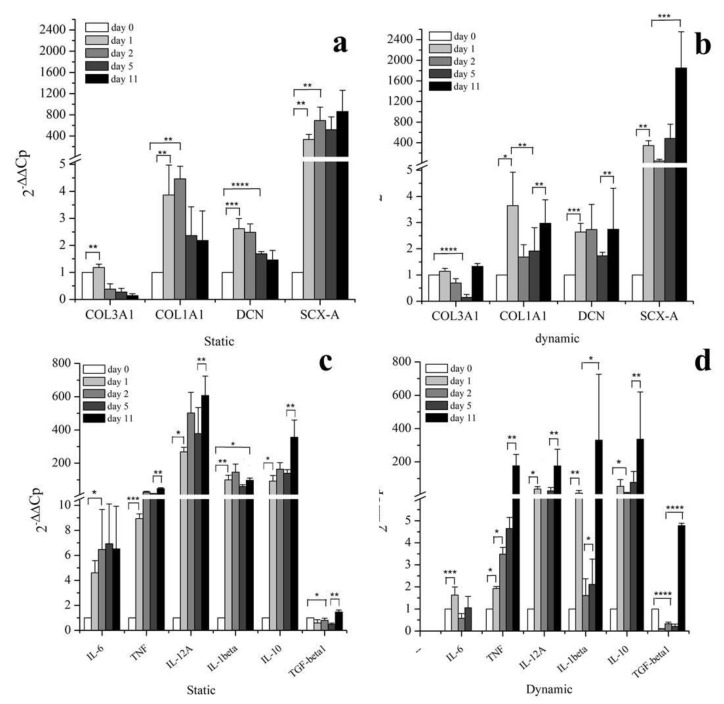
Gene expression profiles of tenogenic markers and pro-inflammatory and anti-inflammatory cytokines from hBM-MSCs within HY-FIB environment in static (**a**,**c**) and dynamic culture (**b**,**d**) up to 11 days. Days 1, 2, 5, and 11 were selected as time points to study the mRNA levels of positive tenogenic markers (COL1A1, DCN, SCX-A, and TNMD), negative ones (COL3A1) and pro-inflammatory (IL-6, TNF, IL-12A and IL-1β) and anti-inflammatory (IL-10 and TGF-β1) cytokines. Effect of HY-FIB environment on cells behavior was visible along the first two days of culture; a better over expression of tenogenic markers and anti-inflammatory cytokines was observed in dynamic culture at Day 11. hBM-MSCs within HY-FIB at time zero were used as control. * <0.05; ** <0.01; *** <0.005; **** <0.001. N = 3 (biological replicates); n = 3 (technical replicates).

HY-FIB samples were collected at Day 1, 2, 5, and 11 to monitor tenogenic and cytokine marker expression. Time points at Day 1 and Day 2 were added for 3D culture to monitor the effect of HY-FIB on cells behavior alone or in combination with cyclic strain culture. Indeed, in static conditions COL1A1 and DCN both displayed significant upregulation of 3.8 fold (COL1A1) and 2.6-fold (DCN) at Days 1 and 2 before dropping progressively to elevated but non-significant levels (Figure 5a and Figure S1 in Supplementary Materials), confirming an HY-FIB effect on this gene expression in the first days of culture. In dynamic conditions, COL1A1 levels displayed responses similar to the static culture in the first two days but progressively rising thereafter to significant levels (2.9 fold) at Day 11, probably due to strain input. DCN expression levels in response to dynamic culture were to be elevated throughout, achieving significance at Day 11 (3-fold) (Figure 5b and Figure S1 of Supplementary Materials). 

SCX-A displayed significant upregulation (~340-fold) in both static and dynamic conditions at Day 1, suggesting an effect of HY-FIB system, on this gene expression. SCX-A levels were substantially elevated in both static and dynamic condition at all following time points studied, even if a larger and significant increase was observed in dynamic condition; an increase of 800-fold in static and of 1600-fold in dynamic culture conditions was monitored at Day 11 (Figure 5a,b and Figure S1 of Supplementary Materials). Tenomodulin gene expression was also tested by RT-qPCR, but no expression was detected, probably because it is an event occurring during late differentiation [65]. Sustained, significant, downregulation of COL3A1 was observed in either static or dynamic conditions which instead either decreased progressively (static) or decreased through to Day 5 before reestablishing Day 0 levels at Day 11 (dynamic). 

The data suggested an overall effect of the 3D environment on cells behavior clearly visible along the first two days of culture; furthermore, a statistically significant COL1A1, DCN, and SCX-A over-expression was observed after 11 days when mechanical strain was provided (Figure S1 in Supplementary Materials). 

Cytokine transcript expression data is illustrated in Figure 5c,d, for static and dynamic culture, respectively. HY-FIB system has an effect also on cytokines gene expression, as observed in all time points monitored with respect to Day 0, within static culture. Indeed, pro-inflammatory cytokines IL-6 (~6-fold), TNF (~10-fold), IL-12A (≤600-fold), and IL-1β (~200-fold) displayed rapid and significant upregulation that was maintained for the entirety of the experimental duration. Anti-inflammatory TGF-β1 on the other hand displayed either no change (Day 11) or significant down-regulation (other time points) while IL-10 exhibited an overall similar profile to Il-1β culminating in marked upregulation at day 11 (~300-fold) (Figure 5c). 

Dynamic culture conditions had a distinct and significant effect on IL-6 with expression levels achieving a peak upregulation of 1.5-fold at Day 1 and decreasing to undetectable levels by Day 11 (Figure S2 in Supplementary Materials). TNF and IL-1β were both gradually upregulated before achieving ~200-fold and ~300-fold, respectively, upregulations at day 11 (compared to 10-fold and 100-fold in static conditions). IL-12 displayed a similar profile of upregulation in dynamic vs. static culture conditions while achieving maximal levels that were 3X less in dynamic. Anti-inflammatory IL-10 expression levels were consistent across both dynamic and static conditions. In contrast to static culture, TGF-β1 was significantly downregulated until day 5, and it underwent a 5-fold increase at Day 11 in dynamic culture conditions (Figure 5d and Figure S2 in Supplementary Materials). 

**Figure 6:**
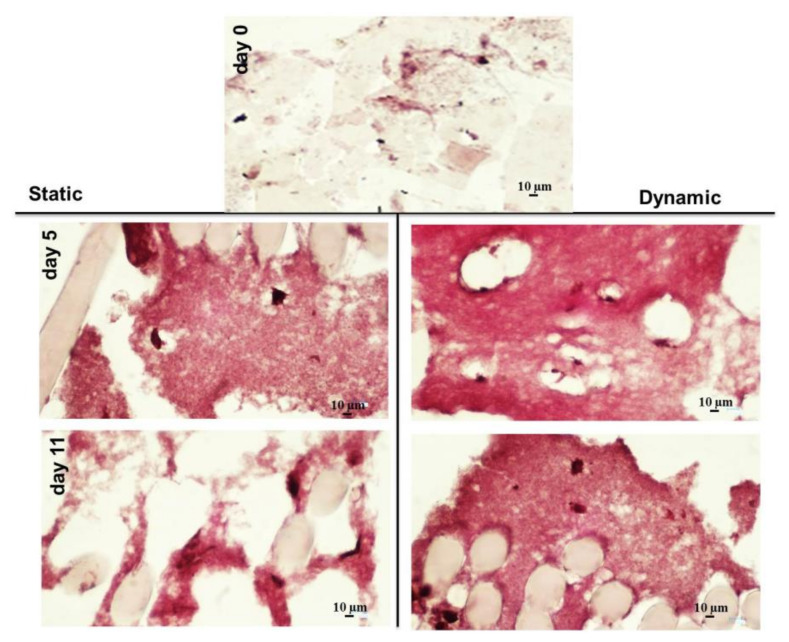
Histology characterization of the overall HY-FIB scaffold structure with Sirius Red staining. HY-FIB scaffolds in both static and dynamic culture at Days 5 and 11 are reported; the overall scaffold structure was stained with Sirius Red for collagen highlighting. Fibrin hydrogel was light pink stained in the sample collected at Day 0. Fibrin matrix was clearly stained in red at Day 5 and 11 in both samples from static and dynamic culture. Less homogeneous scaffold matrix structure and staining was observed in samples taken from static culture.

Histological characterization of HY-FIB scaffold in both static and dynamic culture at Days 5 and 11 are reported in Figure 6; the overall scaffold structure was stained with Sirius Red for collagen highlighting. Despite fibrin hydrogel matrix, collected at Day 0, was only light pink stained, the same matrix was clearly stained in red at Day 5 and 11 in both samples taken from static and dynamic culture. However, a less homogeneous matrix organization and staining was observed in the samples taken from static culture. This data is in agreement with gene expression indications and confirmed that both HY-FIB alone and HY-FIB plus cyclic strain had an effect on cells phenotype commitment.

**Figure 7:**
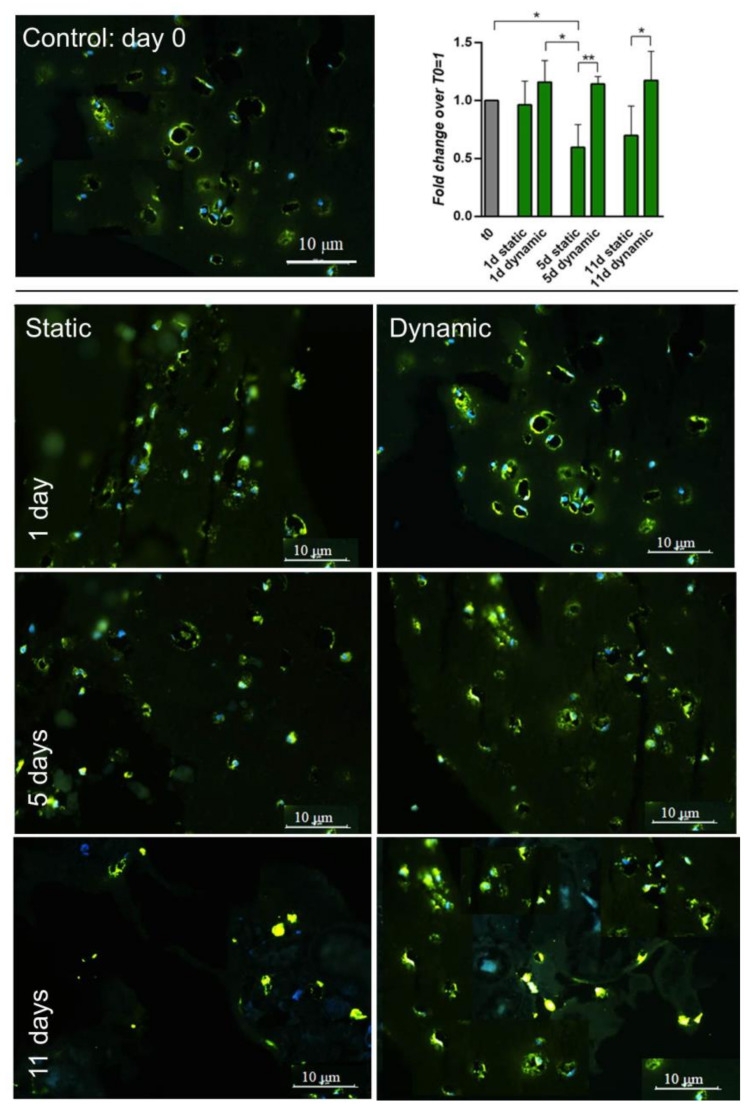
IF and quantitative-IF assays of type 1 Collagen (COL1A1) in 3D static and dynamic culture of hBM-MSCs for 11 days. At day 1, holes within the hydrogel structure are evident nearby cells surroundings. These areas were progressively filled by COL1A1 protein (stained in green) and it happened more uniformly during the dynamic cultivation (see day 11). Fluorescence quantification by ImageJ software, reported in the plot, confirmed the presence of more abundant signal (0.2–0.3 fold changes) in dynamic condition; signal intensity in each time point was normalized by cell number (e.g., by amount of cell nuclei revealed by DAPI staining). * ≤0.05; ** <0.01. N = 3 (biological replicates); n = 3 (technical replicates).

The expression of type 1 Collagen, a tenogenic matrix-associated marker, was monitored by immunofluorescence over the culture time (see Figure 7). At day 1, empty areas surrounding the cells are present, probably due to absence of uniform fibrin hydrogel. These spaces were then progressively filled with the protein, presumably via secretion into the extracellular environment. The level of staining observed under static conditions decreased after Day 1 and was maintained at ~50% of original levels thereafter while levels were maintained consistent to Day 0 in dynamic culture. Moreover, in the dynamic condition a more uniform cells distribution was noted throughout the hydrogel matrix, especially at day 11. 

## 4. Discussion

The HY-FIB system is engineered to support delivery of PLGA nanocarriers (PLGA-NCs) within the hydrogel matrix, enabling controlled delivery of specific molecules within 3D environment, e.g., drugs or other biological signals. The active form of hGDF-5 loaded into PLGA-NCs for controlled delivery within HY-FIB environment was investigated in a previous study [53]. Here, we investigated the effect of the HY-FIB 3D environment (hyaluronate band + PLGA carriers + fibrin gel) on hBM-MSCs tenogenic and cytokine marker gene expression in both static and dynamic, mechanical, input scenarios. We adopted the previous HY-FIB configuration including PLGA-NCs, but on this occasion, we delivered an inactive form of hGDF5. In this manner, the biochemical input provided by the growth factor was excluded, but the complete HY-FIB configuration was maintained, and we were thus able to investigate the impact of mechanical input alone.

HY-FIB braided fibers enabled a defined mechanical stimulation of 9 × 10^−2^ Pa provided to hBM-MSCs during the 4h/day dynamic culture regime. The mean shear stress was calculated by FEM modeling [62], assuming a system homogeneous behavior at a density of 1050 kg/m^3^ and Young’s modulus of 4.56 Mpa [53]. A Poisson ratio value of 0.25 was adopted as described elsewhere [62]. Further mechanical inputs with different intensities and durations were not investigated, not being the aim of the present work. Stress values resembling reduced physiological activity, similar that the ones used here, have been reported to direct stem cell commitment to a tenogenic phenotype [31,36,66].

COL1A1 is the major component of tendon tissue (75–85% of the dry mass of tendon), and is responsible for its mechanical strength [64]. In the static group, COL1A1 showed a ≥3-fold upregulation during the first and second day of cultivation. These data seem to suggest an overall effect of the 3D environment on cells behavior. COL1A1 expression was progressively reduced to a 2-fold upregulation at Day 11, in static environment. In dynamic conditions, its mRNA levels showed similar behavior during the first two days of cultivation (an increase up to 3-fold-changes, then reduced at Day 2). However, its expression was subsequently increased again to 2.8-fold at Day 11. Decorin (DCN), a small leucine-rich proteoglycan implicated in the regulation of fibrillogenesis, is a fundamental component of the tendon extracellular matrix (ECM) [67]. Compared to the static condition, a significant enhancement, up to 2.5-fold, of the mRNA level of DCN was shown when hBM-MSCs were cultured for 11 days with mechanical stimulation.

Scleraxis-A (SCX-A) is a neotendon marker, expressed in pro-tendon sites in the developing embryo. Specifically, SCX-A is a tendon-specific basic helix-loop-helix transcription factor responsible for the transition of MSCs into tendon progenitors [68]. We observed substantial increases in SCX-A expression, up to 800-fold in static and 1600-fold in dynamic conditions after 11 days, demonstrating a stimulatory effect via the 3D system organization and consistent with previous observations [69,70,71].

COL3A1 mRNA level seems to be downregulated after 2 days in the static group and after 5 days in the dynamic group. Its downregulation can be considered a positive indication of proper cell differentiation; indeed, it seems that COL3A1 is the main responsible of fibrotic and scarred tissue arrangement and has been consistently reported at the rupture site of human tendons. [64]. 

From both histology and immunofluorescence assays, we noted that the area surrounding the cells was progressively filled by type 1 Collagen and, at Day 11, the extracellular matrix seemed to undergo remodeling (Figure 6 and Figure 7). Moreover, in dynamic conditions a more homogeneous cell distribution within the hydrogel matrix was observed in the IF images. These findings support the concept that 3D cultivation provides cues to the hBM-MSCs, and that dynamic signaling enables the adoption of a more uniform behavior including type 1 Collagen protein deposition in the externally available space of the fibrin hydrogel. A near total absence of type 3 Collagen was found, except for a very small fluorescence signal at day 1, in both static and dynamic conditions (data not shown). These data suggest that tenogenic commitment of hBM-MSCs cultured within HY-FIB environment may be enhanced when dynamic stretching is applied.

MSCs secrete a variety of cytokines and growth factors that promote cell recruitment, migration, proliferation, and differentiation. MSCs are also immunomodulatory, which may allow them to exert beneficial effects on the local immune cell population at the site of muscle injury [72]. To better understand the hBM-MSCs inflammatory response when cultured within HY-FIB, cytokine expression was monitored along the 11 days of culture. The balance between pro- and anti-inflammatory soluble factors in the tendon healing process exerts a major impact on successful resolution of inflammation [73]. Recent analysis of tendinopathy biopsies showed a distinct inflammatory infiltrate in the initial phase of tendinopathy with a high content of pro-inflammatory factors such as IL-6, TNF-α and IL-17 [74]. 

To exclude a role for ihGDF5 in cytokine expression induction we evaluated their expression in hBM-MSCs undergoing 2D planar cultivation as a negative control. Indeed, in disc degeneration models using in vitro three-dimensional cultures, human annulus cells display increased expression of pro-inflammatory cytokines, such as IL-1β and TNF-α, while exposure to TNF-α and IL-1β resulted in significant downregulation of GDF-5 [75]. Therefore, it is plausible that the GDF-5 may upregulate the expression of pro-inflammatory genes in hBM-MSCs leading to the maintenance of an autocrine feedback. However, when the ihGDF-5 was added, no statistically significant expression of pro-inflammatory cytokines was observed; therefore, ihGDF-5 did not exert any effect on cytokines expression. 

The addition of PLGA-NCs enabled an informed analysis of the inductive role of the HY-FIB overall structure. Previous studies have noted that cytotoxicity of SEE-fabricated PLGA-NCs on blood mononucleate viability, monitored with MTT assay [76], was not affected after either 24 or 48 h. Here, the overall HY-FIB system (loaded with SEE fabricated NCs) does not evidence any toxic effect on hBM-MSC cultivated within it for 11 days, providing an indirect indication about SEE technology as suitable process for biomedical carriers production.

In general, we observed that pro-inflammatory gene expression was higher in static than in dynamic conditions at all-time points. On the contrary, the anti-inflammatory cytokines IL-10 was consistently upregulated in both static and dynamic conditions; while TGF-β1 was downregulated at all the time points tested except day 11, when, it showed a marked increase (4-fold) only in dynamic environment. The described behavior confirmed that MSCs respond to a variety of biophysical cues; indeed, as suggested by Qazi et al., 3D culture of MSCs on biomaterials can promote cell-cell interactions and enhance the paracrine effects of MSCs [77]. Moreover, as concluded by Ogle et al., historically, biomaterial-based therapies to promote tissue regeneration were designed to minimize the host inflammatory response. Recently, the roles that monocytes and macrophages can play in tissue repair have been highlighted. In this context, material properties and their possibility of specific biomolecule controlled delivery has been engineered to achieve a given biological response that can be tuned not only to a better integration with biological systems but also in regulating the inflammatory response [5]. 

The overall and statistically significant balance of pro- vs. anti-inflammatory cytokines expressed by cells provided indications regarding the importance of dynamic culture for 3D in vitro model systems. For instance, IL-6, a well-known pleiotropic cytokine delivered by tissues in response to physio-pathological changes such as physical exercise, infection, and injury, was reported to deeply alter skeletal muscle milieu, by affecting the activity and quality of cellular interactors during tissue regeneration and leading to the fibrotic response [78]. In our 3D model system, IL-6 gene expression was considerably reduced in hBM-MSCs that underwent dynamic 3D HY-FIB cultivation when compared to the same cells cultivated in static condition. 

It is worth of note that there is no specific literature on cytokines response by hBM-MSCs cultivated within 3D scaffold. Almost all published studies described cell-specific differentiation toward a given phenotype, without considering how cytokines expression may be related to a 3D in vitro scaffold system. In this sense, the present investigation is the first study, which suggests cytokines expression as a further variable to monitor cell behavior and reaction when loaded into a 3D in-vitro model. Moreover, improved balance in anti-inflammatory cytokines observed for HY-FIB plus cyclic strain may be considered an indication of better cells response to the 3D in vitro system designed and proposed. 

## 5. Conclusions

The 3D cell culture yielded evidence of type 1 Collagen expression observed by both immunohistology and gene assay. When the same 3D system was cultivated under cyclic strain stimulation, the mechanical input stimulated a statistically significant increase in tenogenic markers expression when compared to the same cells assembled into the 3D system, but cultivated in a static culture. Further studies may involve a deeper understanding of the relation between collagen type I production, cell commitment and mechanical input strain percentage or duration; in this sense, HY-FIB system can be considered a good instrument for this study. The 3D culture system activated also the expression of pro-inflammatory cytokines, and, when cyclic strain was applied, pro-inflammatory cytokine gene over-expression by hBM-MSCs was better balanced against over-expression of anti-inflammatory cytokines. It remains to be determined what the involvement and the immunomodulatory activity of hBM-MSCs are, and the role of implantable biomaterials in the stimulation of inflammatory reactions. For instance, the stimulation of local inflammation is reported as an important event in triggering repair in avascular tissues, such as cartilage and tendons [5]. 

On the other hand, the presence of PLGA-NCs within the fibrin hydrogel would allow the delivery of specific biomolecules that may be studied for the ability to further modulate inflammation reactions or promote regeneration/repair events. In this sense, HY-FIB provides a potential strategic approach to address a range of issues via the provision of a tightly controlled in vitro protocol. The 3D scaffold is a potential system to organize the sustained release of different biochemical signals and opens concrete perspectives for developing 3D bioengineered models to understand specific molecular and cellular composition of damaged systems.

## Figures and Tables

**Table 1:** Finite Element Modeling (FEM) parameters used.

Parameters	Value	Unit
Young’s modulus ^1^	4.56	MPa
Poisson ratio ^2^	0.25	-
Density	1050	Kg/m^3^

^1^ HY-FIB Young Modulus was measured experimentally [53] ^2^ Poisson ratio value was taken in the literature [62].

## Supplementary Materials

The following materials are available online at https://www.mdpi.com/2073-4409/9/5/1268/s1, Figure S1: Profiles of positive (SCX-A, COL1A1, DCN) and negative (COL3A1) tenogenic markers between HY-FIB static vs. dynamic culture. * < 0.05; ** < 0.01; *** < 0.005; **** < 0.001, Figure S2: Profiles of pro-inflammatory (IL-6, TNF, IL-12A, IL-1β) and anti-inflammatory (IL-10, TGFβ1) cytokines markers between HY-FIB static vs. dynamic culture. * < 0.05; ** < 0.01; *** < 0.005; **** < 0.001.

## Patents

The SEE technology for nanocarriers fabrication was described in the US Patent US/8628802 B2 Jan 2014. Inventors: Reverchon E., Della Porta G., Continuous process for microspheres production by using expanded fluids. Applicant: University of Salerno.
