# Supplementary material for: Tendon and Cytokine Marker Expression by Human Bone Marrow Mesenchymal Stem Cells in a Hyaluronate/Poly-Lactic-Co-Glycolic Acid (PLGA)/Fibrin Three-Dimensional (3D) Scaffold
Source: Cells. 2020 May 20;9(5):1268. doi: 10.3390/cells9051268 (PMC7291129; doi:10.3390/cells9051268)
Supplement: Supplementary file 1 [file cells-09-01268-s001.pdf]

Supplementary Data

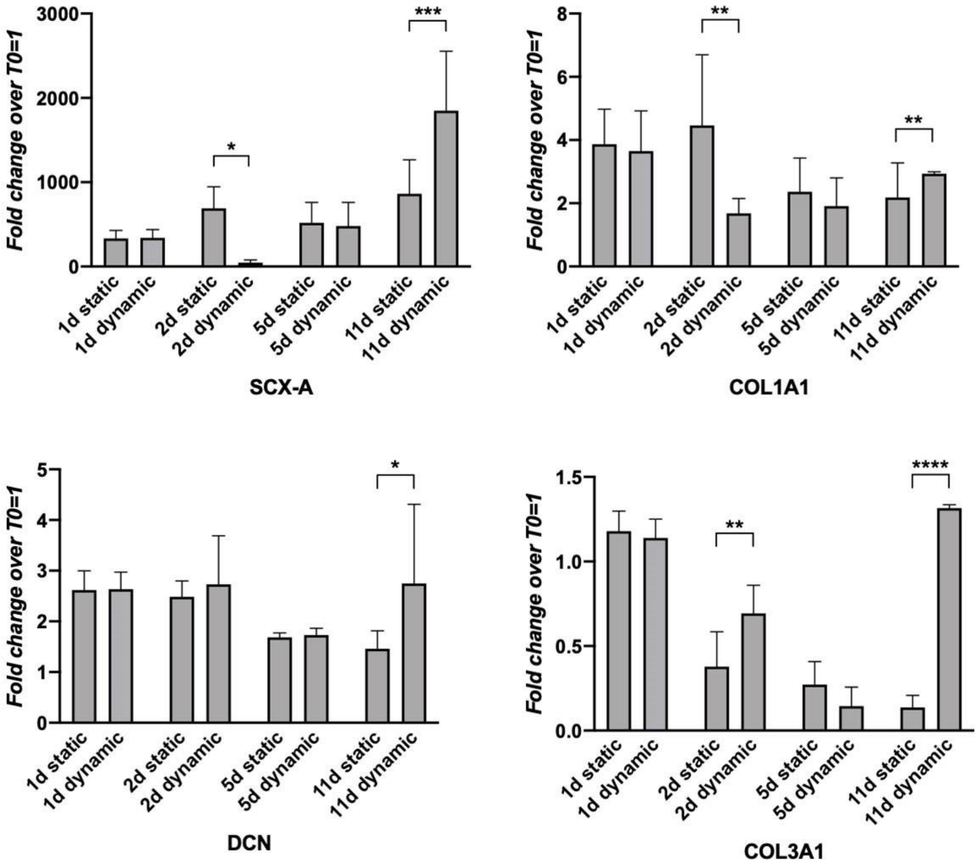

Figure S1. Gene expression profiles of positive (SCX-A, COL1A1, DCN) and negative (COL3A1) tenogenic markers of HY-FIB static vs. dynamic culture. * < 0.05; ** < 0.01; *** < 0.005; **** < 0.001.

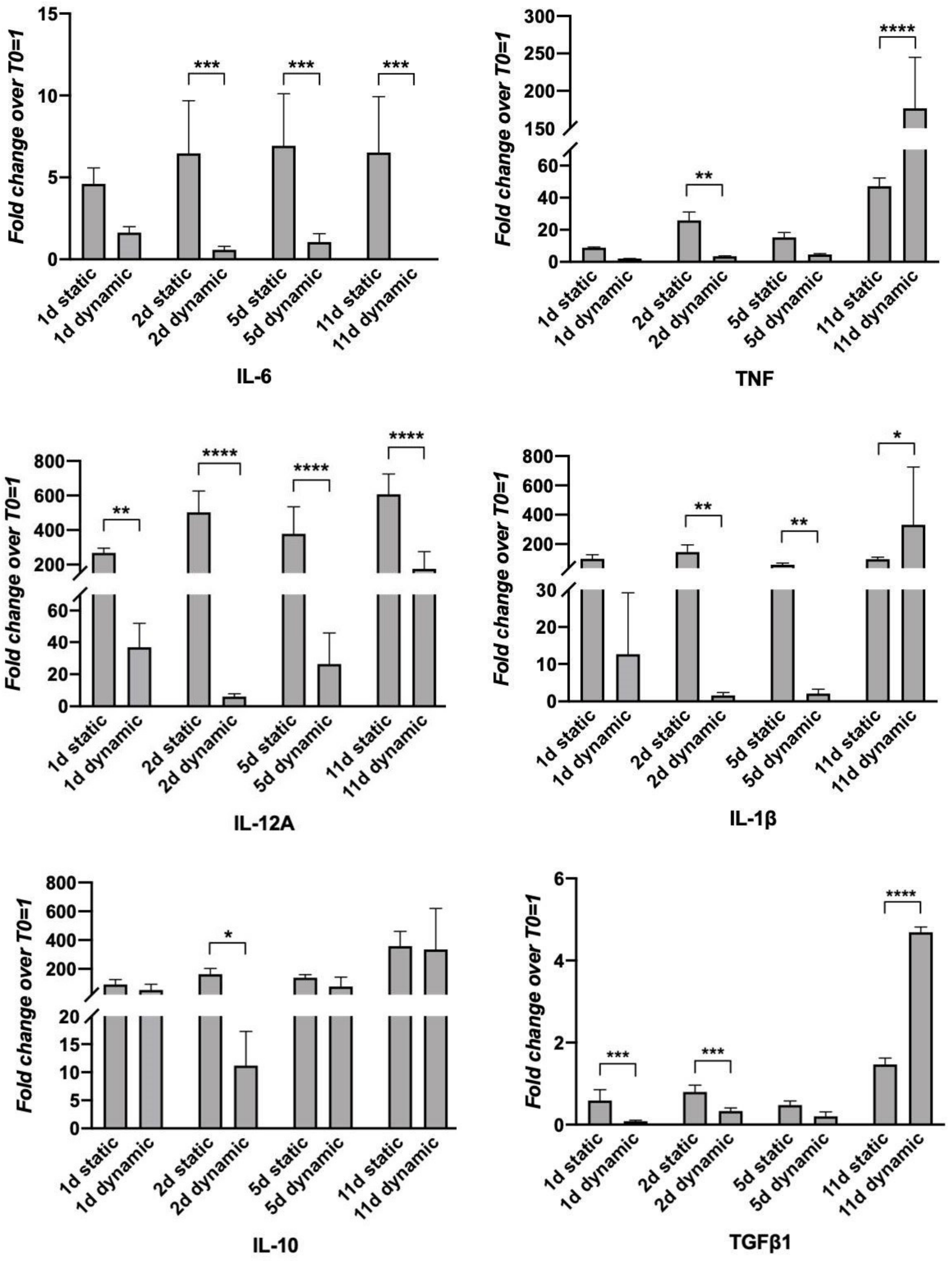

Figure S2. Gene expression profiles of pro-inflammatory (IL-6, TNF, IL-12A, IL-1β) and anti-inflammatory (IL-10, TGFβ1) cytokine markers of HY-FIB static vs. dynamic culture. * < 0.05; ** < 0.01; *** < 0.005; **** < 0.001.
